# Impact of heat stress on milk and meat production

**DOI:** 10.1093/af/vfy026

**Published:** 2018-10-26

**Authors:** Andrea Summer, Isabella Lora, Paolo Formaggioni, Flaviana Gottardo

**Affiliations:** 1 Department of Veterinary Science, University of Parma, Parma, Italy; 2 Department of Animal Medicine, Production and Health, University of Padova, Legnaro (PD), Italy

**Keywords:** cattle welfare, climate change, growth performance, heat stress, milk quality

**Implications**

- In recent years, global warming is a major concern for the agricultural sector.
- Heat stress impairs welfare and productive performance of dairy and beef cattle.
- Different climate conditions have important effects on the organic and inorganic components of milk.
- Heat stress in beef cattle is detectable by homeostatic mechanisms (panting, sweating, and urination) and behavioral alterations such as a reduction in activity, increased water intake, and reduced feed intake.
- Global warming will have significant economic impacts for producers and consumers.

## Introduction

Global warming is a major concern during recent years, and the livestock sector will be one of the most affected segments of the agricultural industry. However, the effects of increasing temperatures on livestock will be different worldwide, based on latitude and farming systems. In addition to the direct effects of heat stress on animal productivity, global warming will also affect soil fertility, water availability, crop yield, and pathogen circulation (Thornton et al., 2009; Nardone et al., 2010). Therefore, in addition to the arid and tropical areas where heat already represents a major constraint, the most affected areas will be those of the subtropical–Mediterranean zones, which are exposed to considerable heat stress for 3 to 5 mo per year (Silanikove, 2000). Other critical factors in these areas are linked to intensive production systems, which are characterized by high farm animal density of high-producing selected breeds and managed in specialized farms. However, the pasture-based system is equally at risk mainly due to the indirect effects of climate change on pasture growth and water availability (Nardone et al., 2010).

Exposure to uncomfortable thermal conditions (due to the combination of high temperature and humidity) overcomes the capacity of cattle to dissipate heat and leads to an increase in body temperature that exceeds the physiological limits (Ronchi et al., 1997). Such condition is called heat stress and impairs the welfare and productive performance of dairy and beef cattle. In this condition, efficiency of nutrient conversion to energy reduces dry matter intake and increases water consumption, and there is a reduction of efficiency in nutrient absorption. In this scenario, cattle performance worsens rapidly (Collier et al., 1982). To evaluate the simultaneous effect of temperature and humidity factors and to assess the risk of heat stress in cattle, the temperature–humidity index is used.

In the case of dairy cows, climate change has an important effect on milk organic and inorganic composition (Mariani et al., 1993, 1998). Climate change also influences the efficiency of cheese manufacturing processes (Sommerfeldt and Baer, 1986), both on cheese yield and on quality and especially for those cheeses produced using raw and not standardized milk.

Heat stress in beef cattle is usually considered less severe than in dairy cattle because beef cattle have a higher average temperature–humidity index threshold due to their lower metabolic rate and lower body heat production (St-Pierre et al., 2003; Nardone et al., 2010). However, beef cattle also will compensate for increased body temperature by homeostatic mechanisms (panting, sweating, and urination) and behavioral alterations such as reduced activity, increased water intake, and reduced feed intake, which will take place preferentially during the coolest hours of the day (Magrin et al., 2017; Marchesini et al., 2018). The changes in feeding behavior will reflect on the efficiency of rumen function up to the onset of metabolic disorders such as ruminal acidosis (Marchesini et al., 2018). The main consequences are generally lower growth rate and reduced fertility of both males and females (St-Pierre et al., 2003).

The economic losses due to heat stress were estimated by St-Pierre et al. (2003) for the major livestock industries in the United States. In the dairy and beef industries, heat stress had a negative economic impact of $897 million and $369 million per year, respectively. Therefore, this article focuses on the main aspects linked to the effects of heat stress on dairy and beef production.

## The Effect of Heat Stress on Milk Production and Quality

### Milk Production

The daily milk yield is highly affected by climate change. The increment of temperature and humidity leads to a significant decrease in milk production (kilogram per day), and this reduction can be easily calculated using the formula proposed by Berry et al. (1964):

Decline in milk production (kg/d) = –1.075 – 1.736 × NL + 0.02474 × NL × THI

where NL is the normal level of daily milk yield (kilogram per day), recorded in the temperature range of 10 to 18 °C, and THI is the daily mean temperature–humidity index.

**Figure 1.**
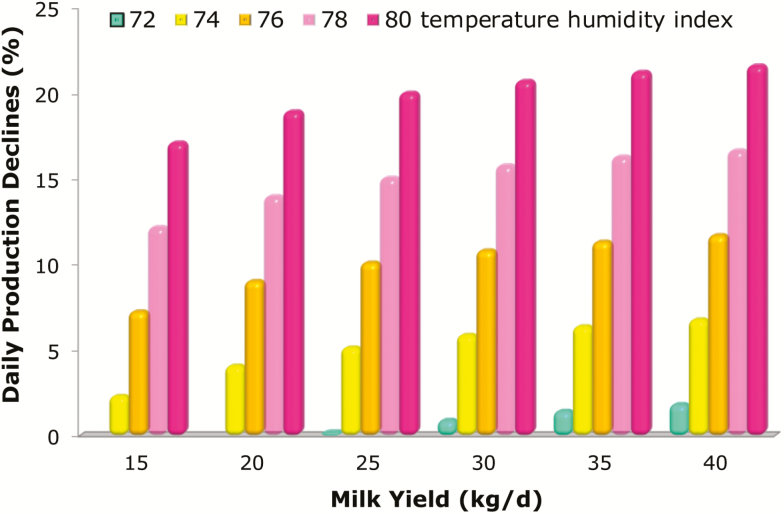
Daily production declines (%) at the increase of temperature–humidity index (Berry et al., 1964).

**Figure 2.**
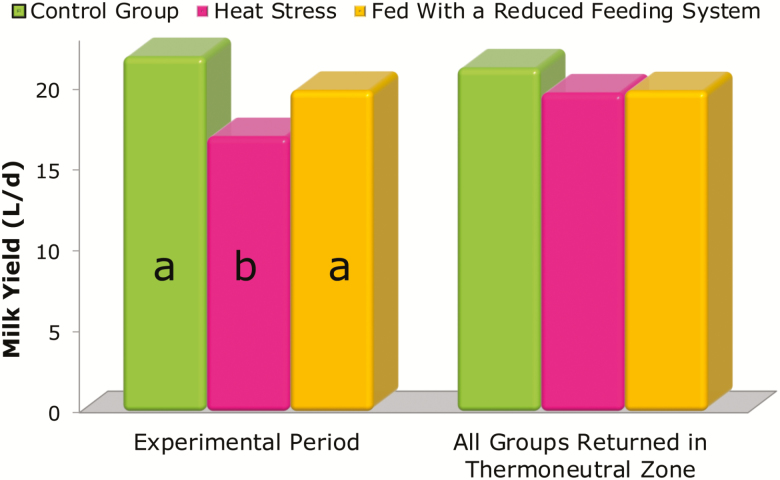
Effect of heat stress and restricted intake (fed with a reduced feeding system) on milk yield. a–b Different letters within period indicate significant differences between treatments (*P* < 0.01; Cowley et al., 2015).

Using this formula, it is clear that daily milk yield (kilogram per day) decreases with the increase of the temperature–humidity index (from 72 to 80), particularly in the more productive cows (from 15 to 40 kg/d). This is even more evident if the decline in milk yield is assessed as percentage of loss (Figure 1). When temperatures move out of the thermo-comfort zone, dairy cows begin to experience heat stress and start to reduce daily milk yield, not because of reduced intake. Accordingly, Cowley et al. (2015) reported that cows subjected to heat stress reduced their ingestion and produced less milk when compared with cows raised in normal climate conditions. In contrast, when cows were fed the same quantity of feed ingested by the heat-stressed group but were not subjected to any heat stress, the decrease in milk production was not significant. In addition, when cows returned to the thermo-comfort zone, milk production increased to the physiological level (Figure 2). To give a practical quantitative evaluation, Bernabucci et al. (2010) reported a loss of 0.27-kg milk per each temperature–humidity index unit incremental change.

### Fat Content

The effect of heat stress on milk fat content is not clear, and controversial results have been reported. Abeni et al. (1993) found lower values of milk fat content when the temperature–humidity index value was higher than 75 (3.46 g/100 g for temperature–humidity index < 75 vs. 3.17 g/100 g for temperature–humidity index > 75, respectively). Bernabucci et al. (2015) reported a marked and significant decrease of milk fat during summer (3.20 g/100 g) compared with the values observed in winter (3.80 g/100 g) and in spring (3.61 g/100 g). Also, Summer et al. (1999) observed a decrease in milk fat content during summer when compared with autumn, ranging from a minimum in June–August (3.36 to 3.38 g/100 g) to a maximum in November (3.67 g/100 g). On the contrary, Cowley et al. (2015) did not find any significant differences for milk fat content between cows in normal conditions or subjected to heat stress.

### Lactose

Milk lactose, the main component of milk after water, is not affected by heat stress of cows. This is confirmed by Abeni et al. (1993) that found milk lactose content not significantly different between cows maintained at temperature–humidity index < 75 and cows maintained at temperature–humidity index > 75 (5.06 vs. 5.10). This result was confirmed also by Cowley et al. (2015).

### Protein, Casein, and Casein Fractions

There are two groups of proteins in milk, caseins, and whey proteins, which are defined by their chemical composition and physical characteristics. Cow’s milk, like that of other ruminants, is rich in caseins and comprises about 77% of total milk protein. Among the various factors involved in the cheese-making process, the role of the protein fraction composition and its seasonal changes for milk coagulation to cheese is globally recognized.

When cows are maintained in conditions of heat stress, both milk protein and casein content tend to decrease. Abeni et al. (1993) reported a decrease of milk protein content when the temperature–humidity index value was higher than 75 (3.02 g/100 g for temperature–humidity index < 75 vs. 2.89 g/100 g for temperature–humidity index > 75, respectively). Cowley et al. (2015) found that cows exposed to heat stress produced milk with less protein than cows housed in comfortable temperature conditions. When cows were fed with a reduced feeding system but were not subjected to heat stress, milk protein showed intermediate values. These results suggest that the decrease of milk protein content is mostly related to a direct effect of heat stress instead of a reduction of feed intake.

Regarding milk casein content, Cowley et al. (2015) found differences between cows raised in comfortable temperatures and the heat-stressed group (28.1 vs. 26.8 g/L, respectively). Milk casein content produced by cows fed reduced amounts of feed (but not heat stressed) was statistically different from that of heat-stressed cows, but not from that of cows raised in comfortable temperatures. This suggests that a reduction of milk casein in the heat-stressed group is due to a direct effect of heat stress and that daily feed intake does not affect milk casein content. In accordance, Bernabucci et al. (2015) found higher milk casein content in winter (2.75 g/100 g) and spring (2.48 g/100 g) with respect to the summer season (2.27 g/100 g).

Milk casein is constituted by several fractions, named α_s1_, α_s2_, β, κ, and γ caseins. The effect of heat-stressed cows on milk casein fractions and their distribution was investigated by Cowley et al. (2015). For those cows subjected to heat stress, an increase of α_s1_ casein and a decrease of α_s2_ casein was observed. The other fractions do not exhibit any difference between groups of cows. Bernabucci et al. (2015) found that milk produced in summer was lower in terms of α_s_ caseins (α_s1_ + α_s2_) and higher of κ casein with respect to the other seasons, whereas β casein was similar. These results could lead to changes in the technological properties of caseins and to a different ability to make cheese.

### Minerals, pH, and Titratable Acidity

Less is known about the impact of cow’s heat stress conditions on milk mineral content and its distribution. Mariani et al. (1993) found significant seasonal variations on the mineral content of milk. These authors observed lower content of milk ash and phosphorus during summer that could be related to the heat stress conditions of cows. Phosphorus has an important role in cheese making, and in some studies, a decrease in phosphorus was related to a worsening of enzymatic milk coagulation.

The climatic conditions in which cows are housed can also affect milk pH and titratable acidity. A correct milk pH, with values around 6.65 to 6.68, and a good milk titratable acidity (the amount of acid compounds in milk), with values from 3.20 to 3.80°Soxhlet-Henkel/100 mL, are essential for an efficient cheese-making process, for high yields of cheese, and for the production of high quality cheeses. Abeni et al. (1993) reported an increase in milk pH and a decrease of titratable acidity when cows were reared at temperature–humidity index values higher than 75. This shows a worsening of these indicators with negative consequences on the production of cheese.

Summer et al. (1999) reported minimum values of milk titratable acidity in August (3.18°Soxhlet-Henkel/100 mL) and maximum values in December and January (3.34 and 3.33°Soxhlet-Henkel/100 mL, respectively).

### Milk Coagulation Properties

Rennet coagulation depends on milk composition and quality. Titratable acidity also has a fundamental role. Casein content and milk salt equilibria (contents of calcium and phosphorus and their repartition between the soluble and colloidal phases) are highly important for this enzymatic process. These factors are particularly important for the Protected Designation of Origin cheeses, especially for long ripened cheeses.

Milk coagulation properties are measured by a lactodynamograph, which previews the addition of a determinate quantity of rennet to 10 mL of milk. At the end of the test, a bell-shaped trace is obtained, from which three coagulation traits are obtained: rennet clotting time, which is the time in minutes between rennet addition and the beginning of milk coagulation; curd firming time (k_20_), which is the time in minutes between the beginning of coagulation and the moment when the bell reaches 20 mm in width; and curd firmness (a_30_), which is the width (millimeter) of the graph at the end of the 30-min analysis.

**Figure 3.**
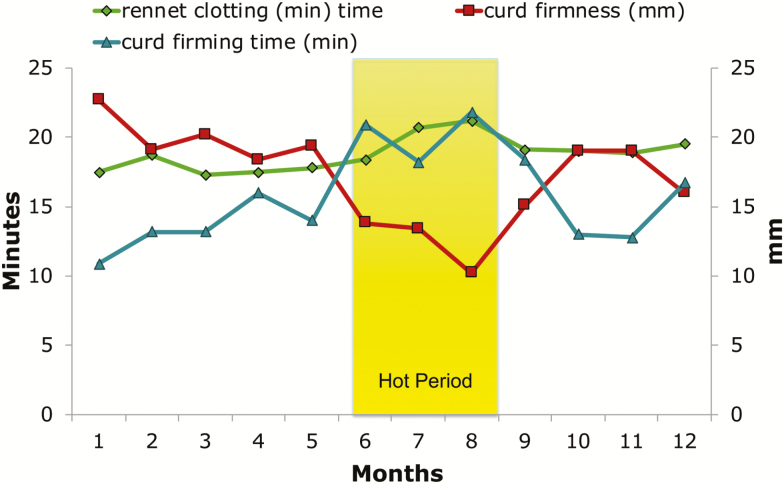
Months of production and Rennet coagulation properties of the milk (Summer et al., 1999). General significance of differences between months was *P* < 0.0001 for all three traits.

Climatic conditions in which cows are housed significantly affect milk coagulation properties. This result is expected because of the strict relationships between coagulation properties and casein content, titratable acidity, and mineral content. The effect on rennet clotting time is marked. Abeni et al. (1993) reported an increased (negative) clotting time when the temperature–humidity index was higher than 75. This result was confirmed by Mariani et al. (1994) that observed the maximum values for clotting time in July (18.97 min) and August (19.42 min) and the minimum in January (15.73 min). In addition, curd firming time is significantly higher when cows are subjected to heat stress. Abeni et al. (1993) observed an increase of curd firming time when the temperature–humidity index value was higher than 75. In the case of a_30_, Abeni et al. (1993) found lower values of curd firmness when the temperature–humidity index value was higher than 75. This result was confirmed by Bernabucci et al. (2015) that found a significant decrease of curd firmness from winter (35.93 mm) and spring (33.60 mm) with respect to summer (21.98 mm). Summer et al. (1999) reported the monthly variation of all the three coagulation traits. In Figure 3, the trends exhibit the increase of clotting time and curd firming time during July and August and a marked decrease of curd firmness during the same months (minimum value of 10.2 mm in August).

Mariani et al. (1994) found during summer months (July and August) the lowest frequency of milk samples with good or discrete coagulation characteristics and the highest frequency of milk samples with bad or anomalous coagulation characteristics. In particular, in August, milk samples with anomalous characteristics reached 10.58% of total analyzed samples. In the same month, milk samples with good coagulation characteristics exhibited the minimum value of 48.69% with respect to the total analyzed samples.

**Figure 4.**
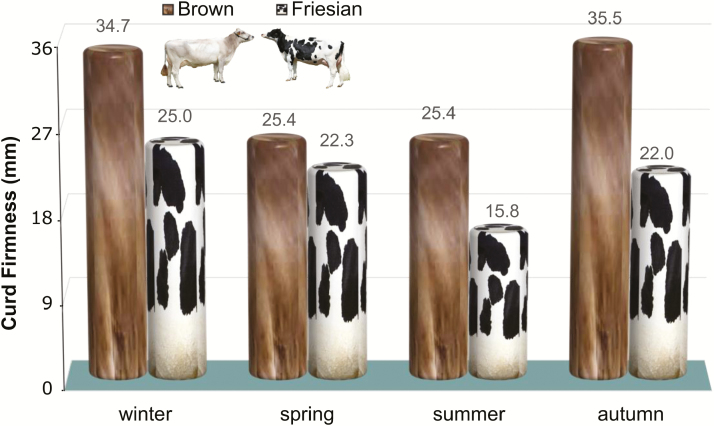
Seasonal trends of curd firmness (millimeter) of Friesian and Brown herd milk (Malacarne et al., 2005).

The response of cows to heat stress is not the same in all breeds. In fact, Malacarne et al. (2005) reported that milk from Italian Friesian cows has a curd firmness value constantly lower than milk from Italian Brown cows (Figure 4). This is due mainly to genetic improvement, particularly on k-casein variant B that was selected in Italian Brown cows. Both breeds showed a decrease during summer, but this decrease was more pronounced for milk from Italian Friesian cows, whereas the value registered for Italian Brown, although lower than values registered in winter and autumn for the same breed, is not different by the value registered in spring.

### Somatic Cells

Milk somatic cells are mainly leukocytes; they increase in milk as a response to an inflammation or infection in the cow’s mammary gland. Heat stress of cows seems to not have any impact on milk somatic cells. Abeni et al. (1993) found that a temperature–humidity index higher than 75 did not affect the production of milk in terms of milk somatic cell content (logSCC 5.12 for temperature–humidity index < 75 vs. 5.31 for temperature–humidity index > 75, respectively). Regarding seasonal variation, both Bernabucci et al. (2015) and Summer et al. (1999) reported an increase of somatic cell content in summer with respect to winter and spring seasons. In fact, if we look at the analyses of milk produced in northern Italy, the content of somatic cells increases in summer.

### Cheese Yield

**Figure 5.**
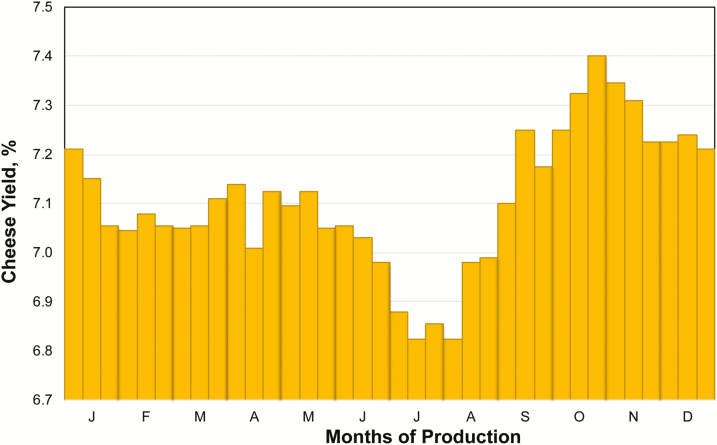
Month of production and Grana Padano cheese yield (%; Mariani et al., 1995).

The characteristics of milk quality produced by cows housed in heat stress conditions lead to a worsening of cheese yield (the amount of cheese obtained by 100 kg of milk). Mariani et al. (1995) reported seasonal variations of Grana Padano cheese yield at 24 h and observed minimum values during the months of July and August, whereas the highest cheese yield was found in the months of October and November (Figure 5).

## The Effect of Heat Stress on Beef Cattle

The adverse effects of heat stress on beef cattle are seen at higher temperature–humidity index compared with dairy cattle. These differences are due to breeds characteristics, production, metabolism, feeding plans, and management systems. In fact, the higher threshold temperature for beef cattle is set at 30 °C with relative humidity below 80% and 27 °C with relative humidity above 80% (SCAHAW, 2001). In contrast to dairy cows, the impact of heat stress on the beef sector is not immediately measurable because it does not reflect on a daily production metric such as milk and may vary depending on several factors. These factors, which will be discussed below, can be summarized by breed, stage of production (e.g., beef cows vs. growing/finishing animals), and production system. Regardless of the cattle category and the production systems, heat stress impairs primarily animal welfare.

### Welfare Concern

As in dairy cows, heat stress in beef cattle is associated with a higher risk of mortality (Thornton et al., 2009). A study by Morignat et al. (2015) found a pooled mortality risk associated with a 1 °C increase above the hot threshold of about 5% for beef cattle. Moreover, the negative effects of heat stress on animal welfare can be observed by changes in animal behavior, which include higher respiratory rates and panting scores, decreased rumination period, and higher frequency of drinking. Affected animals are also more inactive, spend less time eating (especially during the daylight hours), and less time in social interactions (Brown-Brandl et al., 2006; Magrin et al., 2017; Marchesini et al., 2018). These aspects will invariably lead to production losses.

### Cattle Breed

Based on their evolutionary history, different cattle breeds can cope with heat stress with different magnitudes. For example, there is evidence that Braham cattle (*Bos indicus*) can better endure thermal stress than *B. taurus* cattle (Gaughan et al., 2010). Within subspecies, different breeds of *B. taurus* cattle have different levels of heat tolerance. At this regard, a study by Pereira et al. (2014) demonstrated that Limousine cattle cope better with thermal stress and limit the increase in body temperature with lower thermoregulatory reactions than Holstein Friesians. When Limousine is compared with two local Portuguese breeds (Alentejana and Mertolenga), they performed equal to Alentejana and better than Mertolenga in maintaining body temperature stability. Moreover, fatter animals and/or with a heavier hair coat (i.e., higher insulation) and/or darker coated animals (e.g., Angus) are more sensitive to heat (Brown-Brandl et al., 2006; Nardone et al., 2010).

### Stage of Production

The beef cattle sector includes the breeding herd and growing/finishing phases, as well as bulls and heifers, which are generally raised in different locations, with different management practices and sometimes even in different countries (e.g., breeding herds in France and finishing units in Italy). Therefore, the impact of heat stress also differs between the two cattle categories (nursing/breeding cows and growing animals) affecting mainly the reproductive sphere in the case of the cows, and the carcass yield in the case of the beef, without prejudice to the welfare issue in both cases. Regarding the breeding beef cows, the magnitude of the production losses could be relatively small when the breeding season (as traditionally occurs) coincides with a period of low heat stress as in the spring (St-Pierre et al., 2003). The same could happen in the case of finishing beef when the stressful thermal conditions last for a relatively short period and are followed by sufficient time for the animals to recover by compensatory gain. However, it was demonstrated that beef cattle under heat stress had lower growth rates and, when slaughtered during or immediately after this period, had lower carcass weight, lower fat thickness, and worse meat quality in terms of pH, tenderness, and color (Mitlöhner et al., 2001; Nardone et al., 2010; Marchesini et al., 2018).

### Production System

Worldwide, beef cattle are usually raised outdoors and exposed to natural climatic conditions, whereas only a small portion of them are raised in closed housing systems (Nardone et al., 2010). In general, there are three main beef production systems: pasture based (mainly breeding cows), finishing in outdoor feedlots, and finishing in indoor systems. Depending on the system, different factors can influence the occurrence of heat stress in beef cattle.

The pasture-based system is generally adopted for breeding cows, which are maintained under semi-natural conditions, being on pasture from at least spring to autumn (Nguyen et al., 2010). This semi-extensive system allows the animals to freely adopt coping strategies with weather conditions. These animals usually have some access to shade from trees and the possibility to seek water and air movement to cool themselves (Magrin et al., 2017).

The feedlot is an outdoor intensive system in which the factors that enhance heat stress are the confinement of cattle in restricted areas that prevent some of their natural coping behaviors (e.g., migration to cooler areas, seeking the protection of shade, etc.) and the high-energy feeding plan. Therefore, specific measures to mitigate heat stress in finishing beef systems are needed, with regard to feed management and pen facilities. Beneficial effects were reported with the use of restricted feeding plans and ad hoc bunk management that concentrated the feed distribution in the evening and kept the bunks empty during the hottest hours of the day (Mader, 2003). Regarding pen facilities, good results were given by sprinkling cattle or pen surfaces and providing shade (Mitlöhner et al., 2001; Mader, 2003; Nienaber and Hahn, 2007).

**Figure 6.**
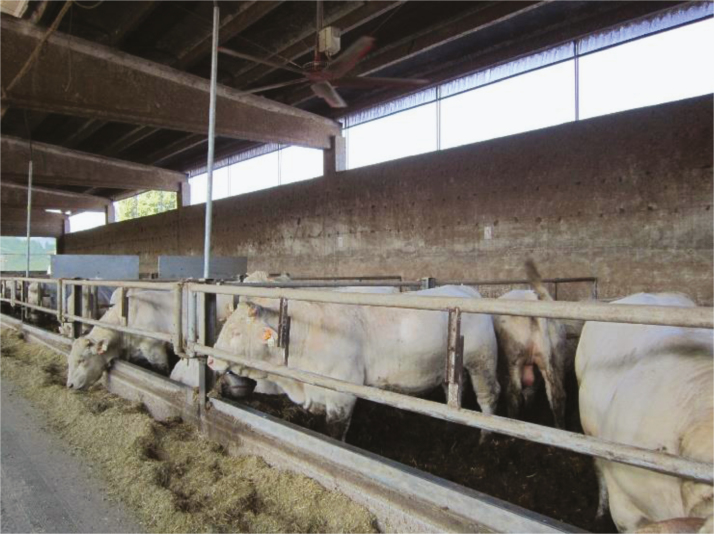
Italian indoor beef cattle housing system.

**Figure 7.**
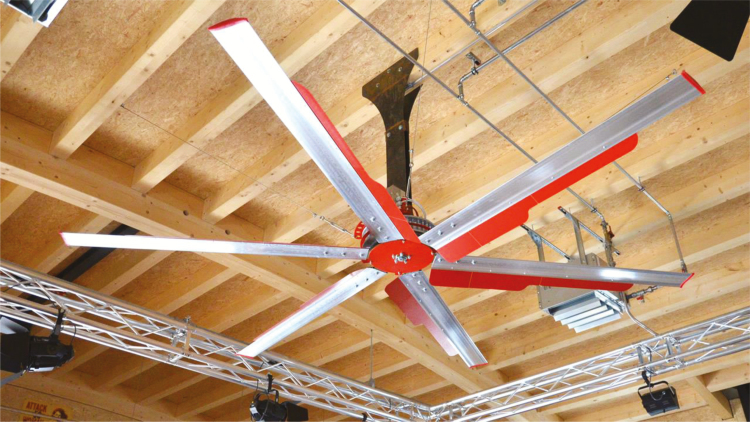
Ceiling fan for the control of the temperature in barns. The fan is fitted with five or six aluminum blades. The diameter of the fan varies from 3 to 5 m depending on the size of the barn.

The indoor finishing system (Figure 6) is largely adopted in Europe, where cattle are often imported from neighboring countries. In this system, young beef cattle coming from pasture-based systems arrive after long trips in a truck and are housed in roofed facilities where they are kept on fully slatted floors or on deep litter (Cozzi et al., 2009). In this case, direct sun radiation is prevented, but animals face other challenges linked to the change in climate conditions and indoor confinement. In fact, besides the extreme temperature–humidity index conditions, cattle are particularly vulnerable to rapid changes in environmental conditions (Nardone et al., 2010). Therefore, factors that can influence heat stress in the indoor finishing systems are the high-energy feeding plan (as in the feedlot) and the environmental and micro-climate conditions of the barns. Hence, heat mitigation strategies in this case include changing the feeding plan (as previously discussed for outdoor feedlots), providing adequate water supply (possibly by additional water stations), and adoption of cooling systems, such as ventilation. Large ceiling fans (Figure 7) gave good results by improving animal welfare, health, and performances and by improving litter (and air) quality due to their ability to increase the circulation of air and dry out the litter (Magrin et al., 2017; Marchesini et al., 2018). Water sprinkling or misting is usually not recommended for indoor systems, as it would increase the slipperiness of slatted floors and the wetness and dirtiness of the deep litter, with negative consequences for the animals. Moreover, misters or sprinklers could limit the efficiency in which animals dissipate heat, especially in cases of already high relative humidity (Magrin et al., 2017).

## Conclusions

Heat stress has considerable effects on cattle welfare and production. In hot and humid climate conditions, dairy cows produce less milk with lower milk quality characteristics, especially those related to cheese-making. In beef cattle, heat stress impairs reproductive performance of nursing cows, decreases growth rate, and worsens meat quality in growing/finishing animals.

In view of the current climate changes, therefore, we need to cope with the increase in global temperature that threatens to affect cattle-derived food production. Consequently, to maintain the quantity and quality of milk and meat products, it will be necessary to modify management systems to adapt to the new climatic conditions. Management options include acting at different levels such as the feeding plan, the selection of resilient animals, and adoption of technological tools (heat mitigations systems, automated systems for feed distribution).

However, adaptation to the new environmental conditions will not be inexpensive because it will require a greater expense in terms of energy consumption (in the indoor, intensive, finishing systems) or lower production (especially in pasture-based systems) due to the restriction of available feed and water resources. Therefore, the effort for maintaining good animal welfare conditions and acceptable levels of production and quality will inevitably reflect an increase in production costs per kilogram of end product.

## About the Authors

**Andrea Summer** is professor of Animal Science in the Department of Veterinary Science at the University of Parma. His main research focuses on the influence of genetic factors on milk quality and milk cheese-making properties, effect of somatic cell counts on milk qualitative characteristics, and the role of minerals in cheese-making. He also characterized milk from different species such as yaks, horses, donkeys, and wild ungulates. He is an expert in the production of Parmigiano-Reggiano cheese and is a member of the control committee of its production. He coordinates the laboratory “Quality of Milk and Dairy Products” at the University of Parma. He is President of the Mastitis Council Italia and of the Italian Dairy Science Association and a member of the Italian International Dairy Federation. Corresponding author: andrea.summer@unipr.it

**Isabella Lora** is a researcher in the Department of Animal Medicine, Production and Health at the University of Padova. She is a veterinarian whose main interest is in cattle management and welfare. She conducted her PhD on colostrum management for dairy calves and passive immunity transfer and her research also studied the welfare of beef and dairy cattle in small-scale farms. She is involved in a research project to improve dairy and beef cattle resiliency according to the management system and environmental challenges.

**Flaviana Gottardo** is professor of Farm Animal Husbandry and Welfare at the University of Padova, Department of Animal Medicine, Production and Health. Her research focuses on farm animal behavior and welfare. She studies optimization of management systems to improve the welfare of dairy and beef cattle and pigs and monitors animal health and welfare at the farm and at the slaughterhouse. She is a scientific coordinator of several national projects and she is the scientific leader of University of Padova research group partner within European projects (Horizon 2020 and Susan EraNet).

**Paolo Formaggioni** graduated in Chemistry and later in Food Science and Technology at the University of Parma. He obtained a PhD in Animal Production, Quality and Food Safety from the University of Parma. Since 1998, he has been characterizing milk for cheese making with particular reference to the protein and mineral components of milk. Since 2015, he has been teaching the course “Parmigiano Reggiano Cheese” at the University of Parma. He is a member of the Scientific Secretariat and of the Editorial Committee of the scientific journal, *Scienza e Tecnica Lattiero-Casearia*.
